# Ephaptic synchronization as a mechanism for selective amplification of stimuli

**DOI:** 10.1186/1471-2202-15-S1-P87

**Published:** 2014-07-21

**Authors:** Aman Chawla, Salvatore D Morgera

**Affiliations:** 1 Department of Electrical Engineering, University of South Florida, Tampa, FL33620, USA

## 

We propose a mechanism for the function of choice in attention and awareness, whereby we refer to the faculty which allows the organism to choose the more significant events from a field of possibilities. We show how ephaptic coupling between sensory neurons in say, the olfactory system, allows for the choice of the information theoretically more significant events from the field of stimuli presented to the organism. We conclude that ephaptic coupling could have a role in organization of the information stored in and processed by the brain.

## Introduction

In this paper we present a very simple information theoretic [1] analysis which shows that if independent stimuli are presented to two adjacent ephaptically interacting [2] axons with differing conduction velocities, then the output at the ends of these axons is precisely the stimulus with the greater entropy.

## Methods

The key tool that we use is very basic information theory. We rely on the concept of temporal coding of information in a neuron wherein information is encoded in the precise time at which an impulse is received at the output of an axon. We consider two parallel ephaptically coupled neurons, stimulated independently, and consider the entropy of the temporally coded input random variables.

## Results

Upon performing information theoretic analysis it is found that at the output of the two coupled axons is delivered a new random variable whose entropy is greater than or equal to the larger of the two entropies that *would* be conveyed by the ephaptically *non*-interacting axons. Thus ephaptic coupling acts as an entropy *amplifier* or at the very least, a greater-entropy-*selector*. It delivers at the destinations copies of a new random variable whose entropy is at least as large as the larger of the two entropies that were input into the system. This indicates that it is the entropy of a stimulus presented to a neuron that may be fundamental in what might *matter* to the nervous system, rather than the precise random variable that bears that entropy.

## Conclusions

Ephaptic coupling appears to have illuminated for us the mechanism of all choosing or selecting activity in the mammalian nervous system, where one stimulus (or event or memory or image) is preferred over another. It suggests that we (the mammalian nervous system) simply choose that which is conveying more information (in the technical sense of information theory) to us and store it up or further process it. Via the channel coding theorem this implies that the channels in the brain must have capacities greater than the largest of possible input entropies that are presentable to the organism. Brains might thus be organizationally adapted to the information that they are required to deal with.
